# Performance of Holstein cows subjected to different cooling sessions during subtropical summer

**DOI:** 10.5713/ab.21.0284

**Published:** 2021-10-29

**Authors:** Musa Bah, Khalid Javed, Talat Naseer Pasha, Muhammad Qamer Shahid

**Affiliations:** 1 Department of Livestock Management, University of Veterinary and Animal Sciences, Lahore 54000, Pakistan; 2 School of Agriculture and Environmental Sciences, University of The Gambia, Serrekunda, The Gambia; 3 Department of Animal Nutrition, University of Veterinary and Animal Sciences, Lahore 54000, Pakistan

**Keywords:** Dairy Cows, Heat Stress, Sprinkler Cooling Sessions, Water Usage

## Abstract

**Objective:**

This study aimed to determine the effect of different cooling sessions (CSs) as a water conservation strategy on physiological, and production responses and welfare in Holstein Friesian cows during subtropical summer in Pakistan.

**Methods:**

Twenty-one cows were subjected to three CS in a completely randomized design. The treatments were: i) eleven hours continuous cooling with sprinklers - control (CNT), ii) four CS, and iii) two CS. The CNT represented the practices of the commercial dairy farms in the area, while the other CSs were used as water reduction strategies. Each CS lasted for 1 h with a 12 min cycle (3 min water on and 9 min off) with a sprinkler flow rate of 1.25 L/min.

**Results:**

The average temperature humidity index of the shed and the outside open area were 81.9 and 82.5, respectively. The results showed that both physiological responses were highest in the 2CS group followed by the CNT and the 4CS (p = 0.001). The CNT and 4CS groups had similar milk yield (p = 0.040). The 4CS group had more lying and eating times than the CNT and 2CS groups (p = 0.000). The cortisol level in the 2CS group was 2.0 and 2.2 μg/dL more than the CNT and the 4CS groups, respectively (p = 0.000).

**Conclusion:**

In conclusion, the 4CS was more efficient in cooling the cows and had better welfare, as it yielded similar milk yield, and better physiological responses than the CNT despite using 90% less water.

## INTRODUCTION

Holstein Friesian cows are well known for their high milk yield. They are getting popular in the commercial dairy sector of Pakistan. However, heat stress is a major challenge for these animals due to the long summer season with high ambient temperature and relative humidity (RH, %) in the area. High investments in terms of energy and water are required to cool these cows during summer.

Spray cooling provided either in the holding pen or at the feed bunk is common because it lowers the body temperature and respiratory rate [1,2] and improves feed intake and milk yield in hot conditions. In recent years, research has been focused on optimizing water use due to the increasing concern of depleting groundwater resources [3–5]. Different water reduction strategies are being used in cooling dairy animals including the effect of the sprinkler flow rate [2–4], duration of the spray [3,6,7], and droplet size [8]. Intermittent cooling sessions (CSs) could be another water reduction strategy. In Punjab, Pakistan, traditionally continuous showering from morning to late evening is done in the corporate dairy sector. In a preliminary survey, we found that on average 840 L of groundwater is used to cool a single Holstein cow per day during summer (unpublished data). This is a huge amount of groundwater being used for cooling dairy animals in this area. Pakistan is projected to become water scarce in 2030 [9], hence the judicious use of this paramount resource is very important. The declining levels of groundwater due to climate change [10] makes water-use efficiency an important issue for sustainable livestock production across the globe.

Limited data are available to compare the efficiency of reduced CS with the traditional method of continuous sprinkler cooling in dairy cows. We hypothesized that the latter would be using more water and might be less effective in cooling cows compared to the former. The objective of this study was to determine the effect of different CS on the physiological, production, and behavioral responses and welfare in Holstein Friesian cows during subtropical summer in Pakistan.

## MATERIALS AND METHODS

### Animals, housing, and management

The present study was conducted at the Dairy Animals Training and Research Center, the University of Veterinary and Animal Sciences (UVAS), Lahore, Ravi Campus, Pattoki, Pakistan (31_03043.9” N 73_52036.1” E) during summer (August and September 2019). All the experimental procedures were approved by the University’s ethical review committee of the UVAS.

Twenty-one lactating Holstein Friesian cows with average daily milk yield 15.9±4.0 kg, days in milk 225.1±47.5, parity 2.6±0.7, body weight 597±7.7 kg, and age 7.2±3.1 years (mean ±standard deviation) were enrolled for the study. The cows were housed in the southern pen of a naturally ventilated freestall shed. The pen was 50 m long (east-west) and 13.5 m wide with the ridge and eve height of 12 and 7 m, respectively. A polyvinyl water pipe (5.08 cm diameter) at a height of 1.9 m was installed along the feed bunk having sprinkler nozzles for showering. The sprinkler nozzles were fitted with a solenoid valve on the water pipe at 2 m apart having 180-degree radius and angled to spray water at the back of the cows.

The pen was divided into three partitions using galvanized steel pipes. Each partition had at least 16 freestalls. The sprinkler nozzles adjacent to the partitions were removed to avoid the spray drift from one group to the other. The cows had 24 h access to the outside open area adjacent to each partition. The pen had industrial fans (Model FS-75; Bilal Electronics, Lahore, Pakistan). The cows were fed a total mixed ration that consisted of 88.9% oat silage and 11.1% concentrate. The concentrate consisted of 33.5% maize grain, 25% canola meal, 30% wheat bran, 10% molasses, and 0.5% premix and lime. Water was provided *ad libitum*. Milking was done in a 6×6 herringbone milking parlor (GEA Farm Technologies, Bönen, Germany) at 0600 and 2000 h.

### Experimental design

The cows were randomly divided into three groups of 7 cows in each group and assigned to three CS in a randomized control design. The CSs were: i) eleven hours continuous cooling with sprinklers from 0700 to 1800 h, control (CNT); ii) four CSs from 0700 to 0800 h, 1000 to 1100 h, 1500 to 1600 h, and 1700 to 1800 h (4CS); and iii) two CSs from 0700 to 0800 h and 1500 to 1600 h (2CS). The CNT represented the practices of the commercial dairy farms in the area. The 4CS and 2CS were used as water reduction strategies. Each CS lasted for 1 h with a 12 min cycle with 3 min water on and 9 min off. The sprinkler flow rate of 1.25 L/min was used as it efficiently cooled buffaloes [4] and Holstein Friesian cows [11]. The experiment lasted for two months (August and September 2019) with a one-week adaptation period.

### Water use and meteorological measures

The volume of water used in cooling for each treatment group (7 cows) was calculated using the following formulae:


Volume of water=3 min ×No. of Cycles per hour (5)×Sprinkler flow rate (1.25 L/min)×No. of nozzles (4)×No. of cooling hours/sessions(
Table 1)

The temperature and humidity data of the shed and the outside open area were taken at four time points (0600, 1300, 1500, and 1800 h) daily using a digital thermo-humidity meter (HTC1, Shenzhen, China). The meter was hung 2.4 m above the ground in the middle of the shed and at the same height in the outside open area. The readings were grouped into three categories as afternoon (the averages of 1300 and 1500 h), morning (0600 h), and evening (1800 h). The following equation was used to calculate the temperature humidity index (THI) [12]:


THI=(1.8×T°C+32)-[(0.55-0.0055×RH)×(1.8×T°C-26)],

where T°C = dry bulb temperature in degrees Celsius.

### Physiological and production measures

Rectal temperature (RT) and respiration rate (RR) were recorded of all cows between 1300 to 1400 h. The recording measuring devices and methods used were similar to those of [4].

Milk yield of individual cows were recorded from meters installed at the milking parlor. Feed intake data were recorded as group data and presented as dry matter intake (DMI)/7 cows. Milk sampling and analysis were done for percentages of protein, fat, lactose, total solid, and solid not fat using a portable milk analyzer (model: Lactoscan Standard; Milktronic Ltd, Nova Zagora, Bulgaria). Body weight and body condition score (BCS) were taken fortnightly.

### Behavioral measures

The 24 h feeding, standing, and lying time behavioral data on individual cows were collected using Nedap CowControl system (NEDAP, Groenlo, Netherlands). Hygiene score was done according to Hughes [13].

### Blood metabolites measures

Blood was collected and centrifuged at 5,000 rpm for 5 min to collect the serum, which was stored at minus 20°C for further analysis. The serum was analyzed for glucose (GLUCOSE, 23503; Biosystems, Barcelona, Barcelona, Spain), blood urea nitrogen (BUN) (BUN, 21516; Biosystems, Spain), and cholesterol (12505 CHOLESTEROL; Biosystems, Spain) using colorimetric kits with the help of a spectrophotometer (Epoch2; BioTek, Winooski, VT, USA).

### Statistical analysis

All the statistical analyses were carried out using SAS (SAS University Edition: SAS 9.4M6 Institute Inc., Cary, NC, USA). Data were assessed for normality according to the Shapiro-Wilk test analyzed using the Univariate Procedure of SAS and then subjected to repeated measures analysis of variance using Mixed Procedure of SAS. The least square means were separated using the PDIFF option with Tukey’s adjusted p-values. Differences were considered significant at p≤0.05 and tendencies at p<0.10.

## RESULTS

### Water use and meteorological measures

On average, the CNT group used 3,000 and 3,150 L/d more water to cool a group of 7 cows compared to those in the 4CS and 2CS groups, respectively (Table 1).

The average values of temperature, RH, and THI inside the shed and outside open area are summarized in Table 2. The average afternoon temperature and THI were 5.3°C and 3.9, higher than the morning, and 2 and 2.6°C, respectively higher than in the evening. The average afternoon outside temperature and THI were higher in the afternoon than in the evening. However, the morning temperature and RH outside were 0.2°C lower and 2.4% higher, respectively than the inside shed (Table 2).

### Physiological and production responses

The different treatments significantly affected the physiological responses (Table 3). The cows in the 4CS group had the lowest body temperature (38.7°C) and respiratory rate (63 breaths/min), and the highest was recorded in the 2CS group (39.3°C and 79.2 breaths/min; p = 0.000; Table 3).

The daily milk yield was 1.1 kg/d more in the 4CS than the 2CS group (p = 0.040; Table 3). However, the 4CS and CNT had statistically similar milk yield (11.9 vs 11.0, respectively; standard error (SE) = 0.33 kg, p>0.05). Likewise, the CNT and 2CS also had similar milk yield (11.0 vs 10.8, respectively; SE = 0.33 kg, p>0.05).

No difference was observed in the average milk fat% (p = 0.170). The average fat content of the cows was 4.0 (p = 0.170; Table 3). Similarly, no difference was observed in lactose content among the treatments. The cows in the 4CS group had 0.6% more total solids than those in the CNT and the 2CS groups (p>0.05; Table 3).

The group DMI was 6.18 and 4.31 kg/7 cows more in the 4CS and CNT groups, respectively than the 2CS group (p = 0.003; Table 3). The treatments did not have any influence on body weight and BCS of the cows (Table 3). The average BCS and body weight of cows during the study period were 2.68±0.07 and 591±24.7 kg (Table 3).

### Behavioral responses

The cows in the 4CS had 44.5 and 30.6 min/24 h more eating time compared to those in the CNT and 2CS groups, respectively (p = 0.000; Table 4). The CNT and 2CS had similar eating times (311.4 vs 325.3 min, respectively; SE = 8.86 min/24 h; p<0.05). It was observed that the 4CS group had 50.4 and 78.2 min more lying time than those in CNT and 2CS groups, respectively (p = 0.000). The standup frequencies tended to differ (p = 0.054). The average standup frequency in this study was 10.5. The average number of steps among the different treatments differed, with the highest in the 4CS and the lowest in the CNT group (p = 0.000; Table 4). The 4CS group had a 0.23 hygiene score more than the CNT and 2CS groups (p = 0.000; Table 4). Similar hygiene scores were observed in the CNT and 2CS groups (1.37 vs 1.37, respectively; SE = 0.46; p>0.05; Table 4).

### Blood metabolites

No difference was observed among treatment groups for serum glucose, BUN, and cholesterol (p = 0.603, 0.698, and 0.127, respectively; Table 5). The CNT and 4CS groups had 2.0 and 2.2 μg/dL lower cortisol levels than the 2CS group, respectively (p = 0.000). However, there was no difference in cortisol level between the cows in CNT and those in the 4CS (4.8 vs 4.6, respectively; SE = 0.30; Table 5).

## DISCUSSION

### Water use and meteorological measures

The average volume of water used to cool one cow per day was in the ratio of 20:2:1 for the CNT, 4CS, and 2CS, respectively. This showed a large margin of water usage between the traditional cooling (CNT) and the two water reduction strategies (4CS and 2CS). In instances where cows are kept in tie stall, with one nozzle per animal, the total water usage may be as high as 825 L/day per animal for the same duration of sprinkling instead of the 428.6 L in this study.

The THI is normally used to summate the intensity of heat stress on dairy cows [14]. The high THI values (>84) showed that the cows were under moderate heat stress during the month of August and September. The lower temperature and THI inside the shed than the outside open area could be attributed to the shade provided by steel roof. This study has shown that the peak of temperature and THI in both the inside and outside open area was in the afternoon. The lower THI values during the morning and evening hours indicated that cows had less heat load during the relatively coolers hours of the days. The current temperature and THI pattern could serve as a guide for future showering strategies under similar THI conditions.

The meteorological measures were recorded only for one location i.e., in the middle of the shed and not for individual partitions. As mentioned earlier, there were no physical barriers between the partitions except steel pipes. The measurement of meteorological data for individual partition would be of limited usage because cooling cows by showering does not change the microenvironment of the shed; rather it helps to increase the heat abatement through evaporative cooling [15].

### Physiological and production responses

The presence of high temperatures and high RH interferes with heat abatement ability of cows [16] resulting in heat stress. In this study, the spray cooling using sprinklers was done at the feed bunk. Research has shown that spray cooling provided either in the holding pen or at the feed bunk is commonly used as it lowers body temperature and respiratory rate [7].

The low RT in the 4CS suggested that the CS was able to lower the body temperature of the cows compared to the CNT and 2CS groups. Similar to this study, cows subjected to 8 CSs rather than 5 CSs have been reported with lower RT and RR [6]. This study and that of Honig et al [6] yielded lower RT and RR with increased CSs. Physiological measures are important indicators of animal welfare [17]. The 4CS treatment had a better animal welfare result because it had lower RT and RR of the cows.

The unexpected higher physiological responses of the CNT group compared to the 4CS group could have been influenced due to the location of the treatment group in the pen and the time of physiological data collection. The CNT group was in the eastern partition of the pen, while the 4CS group was in the middle. As the shed was naturally ventilated with open wall from all sides, it allowed direct solar radiation in this partition (CNT group) during the morning hours. The intensity of these solar radiations could have caused an increase in temperature in the pen that might have affected the physiological responses of these cows. Secondly, the physiological responses were taken between 1300 and 1400 h. Although during this period (1300 to 1400 h), the overhang provided shade in all the partitions, the cows in the CNT might have not fully dissipated the heat load accumulated during the early morning hours. Future trials with a modified housing having a curtain on the eastern end of the shed to avoid direct solar radiation during early hours of the day and a more appropriate study design would be helpful to further explain the response to different showering sessions.

In this study, the daily milk yield did not differ between the CNT and 4CS groups. Earlier studies had reported similar results where applying more water resulted in a diminishing return [2,18]. However, the 4CS group had more milk yield than the 2CS. This result agreed with Honig et al [6] who reported that increasing CSs had positive effects when THI is high. The decrease in feed intake had been reported to account for about 50% of the reduction in milk [19], and the remaining due to other physiological mechanisms [20]. The difference in milk yield between the 4CS and 2CS groups could be attributed to both the feed intake and physiological response. In this study, the milk fat did not differ among treatments. The average milk fat in this study was higher than previously reported study [21]. This difference could be attributed to the average milk yield, 32.7 kg/d in that study and 11.2 kg/d in this study. High producing dairy animals had been found to yield less milk components [21].

The overall feed intake of cows was lower because they were under moderate heat stress (THI>84). Considering the production status of cows, 4 to 6 kg difference in a group of 7 cows would be of limited biological value. No effect of treatment on body weight and BCS could be attributed to the fact that all cows were under heat stress and were striving for survival instead of having increased feed intake to put on weight and BCS.

### Behavioral responses

The average eating time of the cows in this study (5.2 to 5.9 h/24 h) was similar to that of previously reported total time spent at feed bunk (5.4 to 5.9 h/24 h; [2]). The average daily lying time in this study was 9.5 h/24 h. Contrary to current findings, Chen et al [2] reported an average lying time of 12.1 h/24 h. The difference in lying duration between Chen et al [2] and this study could be attributed to the difference in temperature and THI; 32.8°C and 78 in their study while 33.1°C and 84.1 in this study. However, the average lying time in this study was higher than reported by Honig et al [6]. The less lying time (8 h/24 h) reported by Honig et al [6] could be due to the movement of the cows from their home pen to the cooling area (holding area of milking parlor), unlike this study where the cooling was done in the home pen. According to Cook et al [22], the lying time of cows in free stalls ranged from 11 to 14 h under thermo-neutral conditions, with thirty percent reduction when temperatures increase. In this study, the 4CS was less than a 30% reduction in lying time and the CNT was within this limit, whiles the 2CS was outside this range, despite the lack of significant difference between the CNT and 2CS groups. This suggested that the 2CS group had more heat load than both the CNT and 4CS groups.

The increased standing time for the CNT group suggested that the cows were benefitting from the cooling effects of the sprinklers. Whereas the 2CS increased in standing time suggested increase in exposing more body surface area for heat abatement to reduce heat load [14,22]. The decreased standing time in the 4CS consequently increased lying time could be associated with reduced heat load. The tendency in more standups in the CNT group could be attributed to the continuous availability of sprinkler flow rates. This could have triggered the cows to go back and forth from freestalls to the sprinklers. The high number of steps in the 4CS could be attributed to the more intermittent showering and feeding as the group also spent more time in feeding (eating). The greater number of steps in the 2CS than the CNT could be attributed to more frequent visits to the water trough either drinking or to benefit from the microclimate of the water trough area [23].

### Blood metabolites

In agreement with this study, similar THI value had been reported to be associated with a significant increase in cortisol level [24]. The average cortisol level in this study was 5.4 which exceeded the normal range (3.8 to 4.4 ng/mL) [25], this indicated that the cows were heat stressed. The relatively lower values of cortisol in 4CS group indicated that the cows in the 4CS group had lower heat load than those in the CNT and 2CS.

## CONCLUSION

This study has provided experimental evidence for reducing the quantity of groundwater to efficiently cool lactating cows in the home pen during summer in a subtropical summer climate. Compared to the CNT treatment, the 4CS treatment yielded better production responses, lower blood cortisol, more cow comfort, and less incidence of mastitis despite using 90.9% less groundwater, and both proved significantly better than the 2CS. This study is the first to demonstrate that 4CS with sprinklers that intermittently deliver 1.25 L/min can provide efficient heat abatement, better udder health, and cow welfare during subtropical summer.

## Figures and Tables

**Table 1:** The different measures of each treatment group with respect to groundwater usage

Measures	Treatments^1)^		

	CNT	4CS	2CS
No. of hours or cooling session	11	4	2
Spray duration (min/h)	60	15	15
Flow rate (L/min)	1.25	1.25	1.25
No. of nozzles	4	4	4
Total volume of water used (L/11 h)	3,300	300	150
Water used/cow (L)	471	43	21

1) CNT, control 11 h continuous cooling with sprinklers from 0700 to 1800 h; 4CS, four cooling sessions from 0700 to 0800 h, 1000 to 1100 h, 1500 to 1600 h, and 1700 to 1800 h; 2CS, two cooling sessions from 0700 to 0800 h and 1500 to 1600 h.

**Table 2:** Summary of the average meteorological measures from August to September 2019

Measures	Morning^1)^			Afternoon^2)^			Evening^3)^		
		
	Means	SD	Range	Means	SD	Range	Means	SD	Range
Inside pen									
Temperature (°C)	27.8	1.4	25.8–29.7	33.1	2.3	27.6–35.6	31.1	1.7	27.8–33.6
RH (%)	87.2	13.1	55.5–96.9	51.2	11.5	40.9–78.5	62.6	8.7	50.9–77.0
THI	80.2	2.0	77.6–83.3	84.1	5.3	78.5–93.9	81.5	1.6	79.0–83.6
Outside open area									
Temperature (°C)	27.6	1.2	25.7–29.6	35.9	2.4	30.6–38.6	31.6	1.4	29.2–33.3
RH (%)	89.6	6.5	79.0–97.4	47.1	7.0	38.9–60.4	61.7	9.4	49.3–75.0
THI	80.4	2.6	76.5–84.0	85.0	2.8	79.4–87.8	82.1	1.7	79.6–85.0

SD, standard deviation; RH, relative humidity; THI, temperature humidity index.

1) At 0600 h.

2) Average of 1300 and 1500 h data.

3) At 1800 h.

**Table 3:** Effect of different treatments on physiological and production responses of Holstein Friesian cows during summer (n = 21)

Measures	Treatments^1)^			SEM	p-value

	CNT	4CS	2CS		
Physiological responses					
Rectal temperature (°C)	39.0^a^	38.7^b^	39.3^c^	0.05	0.000
RR (breaths/min)	69.9^a^	63.0^b^	79.2^c^	1.79	0.000
Production responses					
DMI (kg/7cows)^2)^	80.06^a^	81.93^a^	75.75^b^	3.50	0.003
BCS	2.64	2.77	2.64	0.07	0.322
Weight (kg)	566.5	597.7	608.8	24.7	0.469
Milk yield (kg)	11.0^ab^	11.9^a^	10.8^b^	0.33	0.040
Milk components					
Fat (%)	4.0	4.2	3.9	0.10	0.170
Protein (%)	2.9	3.0	2.9	0.05	0.153
Lactose (%)	4.56	4.80	4.60	0.18	0.130
Total solids (%)	12.2^a^	12.8^b^	12.2^a^	0.08	0.002

SEM, standard error of the mean; DMI, dry matter intake; BCS, body condition score.

1) CNT, control 11 h continuous cooling with sprinklers from 0700 to 1800 h; 4CS, four cooling sessions from 0700 to 0800 h, 1000 to 1100 h, 1500 to 1600 h, and 1700 to 1800 h; 2CS, two cooling sessions from 0700 to 0800 h and 1500 to 1600 h.

2) DMI is for a group of 7 cows per treatment.

a–c Values with different superscripts in a row are significantly different (p≤0.05).

**Table 4:** Effect of different treatments on the behavioral responses and hygiene score of Holstein Friesian cows during summer (n = 21)

Measure	Treatments^1)^			SEM	p-value

	CNT	4CS	2CS		
Eating time/24 h, min	311.4^a^	355.9^b^	325.3^a^	8.86	0.000
Lying time/24 h, min	560.6^a^	611.0^b^	532.8^a^	13.80	0.000
Stand up frequency, No.	11.1	10.1	10.3	0.31	0.054
Number of steps, No.	3,044.3^a^	3,513.1^b^	3,273.5^c^	69.32	0.000
Hygiene score^2)^	1.37^a^	1.60^b^	1.37^a^	0.46	0.000

SEM, standard error of the mean.

1) CNT, control 11 h continuous cooling with sprinklers from 0700 to 1800 h; 4CS, four cooling sessions from 0700 to 0800 h, 1000 to 1100 h, 1500 to 1600 h, and 1700 to 1800 h; 2CS, two cooling sessions from 0700 to 0800 h and 1500 to 1600 h.

2) Hygiene score was done on three body parts of the cow (hind legs, udder, and flank) using a 5–point scale from 1–5 score and the average was used for analysis.

a–c Values with different superscripts in a row are significantly different (p≤0.05).

**Table 5:** Blood metabolites measures of Holstein Friesian cows under different treatments during summer (n = 21)

Measures	Treatments^1)^			SEM	p-value

	CNT	4CS	2CS		
Glucose (mg/dL)	69.1	67.2	69.6	1.75	0.603
Blood urea nitrogen (mg/dL)	14.9	16.1	15.9	0.98	0.698
Cholesterol (mg/dL)	44.1	44.7	58.7	5.65	0.127
Cortisol (μg/dL)	4.8^a^	4.6^a^	6.8^b^	0.30	0.000

SEM, standard error of the mean.

1) CNT, control 11 h continuous cooling with sprinklers from 0700 to 1800 h; 4CS, four cooling sessions from 0700 to 0800 h, 1000 to 1100 h, 1500 to 1600 h, and 1700 to 1800 h; 2CS, two cooling sessions from 0700 to 0800 h and 1500 to 1600 h.

a,b Values with different superscripts in a row are significantly different (p≤0.05).

## References

[1] Kendall PE, Verkerk GA, Webster JR, Tucker CB (2007). Sprinklers and shade cool cows and reduce insect-avoidance behaviour in pasture-based dairy systems. J Dairy Sci.

[2] Chen JM, Schütz KE, Tucker CB (2016). Cooling cows efficiently with water spray: Behavioral, physiological and production responses to sprinkler at feed bunk. J Dairy Sci.

[3] Tresoldi G, Schütz KE, Tucker CB (2019). Cooling cows with sprinklers: Effects of soaker flow rate and timing on behavioral and physiological responses to heat load and production. J Dairy Sci.

[4] Bah M, Rashid MA, Javed K, Pasha TN, Shahid MQ (2021). Effects of sprinkler flow rate on physiological, behavioral and production responses of Nili Ravi buffaloes during subtropical summer. Animals.

[5] VanderZaag AC, Burtt S, Vergé X (2018). Case Study: Water budget of a dairy farm with a tie-stall barn for milk cows and summer pasturing of heifers and dry cows. Prof Anim Sci.

[6] Honig H, Miron J, Lehrer H (2012). Performance and welfare of high-yielding dairy cows subjected to 5 or 8 cooling sessions daily under hot and humid climate. J Dairy Sci.

[7] Tresoldi G, Schütz KE, Tucker CB (2018). Cooling cows with sprinklers: Spray duration affects physiological responses to heat load. J Dairy Sci.

[8] Chen JM, Schütz KE, Tucker CB (2015). Cooling cows efficiently with sprinklers: Physiological responses to water spray. J Dairy Sci.

[9] Mustafa D, Akhter M, Nasrallah N (2013). Understanding Pakistan’s water-security nexus.

[10] Taylor RG, Scanlon B, Döll P (2013). Groundwater and climate change. Nat Clim Change.

[11] Bah M, Javed K, Pasha TN, Shahid MQ (2021). Sprinkler flow rate affects physiological, behavioural and production responses of Holstein cows during heat stress. S Afr J Anim Sci.

[12] Kelly CF, Bond TE, National Research Council (1971). Bioclimatic factors and their measurement A guide to environmental research on animals.

[13] Hughes J (2001). A system for assessing cow cleanliness. In Practice.

[14] Becker CA, Aghalari A, Marufuzzaman M, Stone AE (2021). Predicting dairy cattle heat stress using machine learning techniques. J Dairy Sci.

[15] Butt MA, Bhatti JA, Khalique A, Shahid MQ (2020). Effect of fans and showers on the physiological measures and reproductive performance of Holstein Friesian bulls during subtropical summer. Trop Anim Health Prod.

[16] Allen JD, Hall LW, Collier RJ, Smith JF (2015). Effect of core body temperature, time of day, and climate conditions on behavioral patterns of lactating dairy cows experiencing mild to moderate heat stress. J Dairy Sci.

[17] Polsky L, von Keyserlingk MAG (2017). Invited review: Effects of heat stress on dairy cattle welfare. J Dairy Sci.

[18] Means SL, Bucklin RA, Nordstedt RA (1992). Water application rates for a sprinkler and fan cooling system in hot, humid climates. Appl Eng Agric.

[19] Wheelock JB, Rhoads RP, VanBaale MJ, Sanders SR, Baumgard LH (2010). Effects of heat stress on energetic metabolism in lactating Holstein cows. J Dairy Sci.

[20] Baumgard LH, Rhoads RP (2012). Ruminant nutrition symposium: Ruminant production and metabolic responses to heat stress. J Anim Sci.

[21] Smith DL, Smith T, Rude BJ, Ward SH (2013). Comparison of the effects of heat stress on milk and component yields and somatic cell score in Holstein and Jersey cows. J Dairy Sci.

[22] Cook NB, Mentink RL, Bennett TB, Burgi K (2007). The effect of heat stress and lameness on time budgets of lactating dairy cows. J Dairy Sci.

[23] Schütz KE, Rogers AR, Cox NR, Webster JR, Tucker CB (2011). Dairy cattle prefer shade over sprinklers: Effects on behavior and physiology. J Dairy Sci.

[24] Kim WS, Lee JS, Jeon SW (2018). Correlation between blood, physiological and behavioral parameters in beef calves under heat stress. Asian-Australas J Anim Sci.

[25] Yoshida C, Nakao T (2005). Response of plasma cortisol and progesterone after ACTH challenge in ovariectomized lactating dairy cows. J Reprod Dev.

